# Horse Racing as a Model to Study the Relationship between Air Pollutants and Physical Performance

**DOI:** 10.3390/ani12091139

**Published:** 2022-04-28

**Authors:** Oscar F. Araneda

**Affiliations:** Integrative Laboratory of Biomechanics and Physiology of Effort, LIBFE, School of Kinesiology, Faculty of Medicine, Universidad de los Andes, Santiago 8320000, Chile; ofaraneda@miuandes.cl

**Keywords:** exercise, air pollution, performance, thoroughbred racehorses

## Abstract

**Simple Summary:**

Ambient air contains a mixture of pollutants, the effects of which on animal and human health have been widely described. In contrast, the effects on physical performance are poorly understood, largely due to the difficulty of implementing an experimental model to study this problem. Thoroughbred horse racing involves many animal athletes, of similar genetics, environmental exposure, training, and diet, who participate by breathing varying mixtures of ambient air. This paper presents an analysis strategy based on the homogeneity of the races, the distance, and the design of the track. This paper presents a preliminary analysis in which we observe that the level of performance is decreased by concentrations of PM_10_, PM_2.5_, NO_2_, NO, and CO in the air. Thus, we note that this natural experiment may constitute a model of interest to advance the understanding of the problem.

**Abstract:**

This study proposes the theoretical principles for the selection of a sample of horse races to study the relationship between air pollutants and performance. These criteria were then applied to an original dataset comparing the correlations between these variables obtained in “Handicap” versus “Conditional” type races. Methods: The mean concentration of pollutants during the six hours prior to the race and the speed of the test were determined in 441 official races at a racecourse in Santiago, Chile, during the summer and winter months of 2012. Using layout, track condition and distance (1000, 1100 and 1200 m) as criteria, a homogeneous group of races (“Handicap”; n = 214) versus a heterogeneous group (“Conditional”; n = 95) were compared using simple correlations (Spearman’s test). Results: Race speed was related to greater levels of PM_10_, PM_2.5_, NO_2_, NO and SO_2_ and it was positively related to O_3_, a trend that was observed in the 1000, 1100 m races and in the total “Handicap” group. Similar results were observed only in 1000 m for the “Conditional” group with lower Rho, except for PM_10_ and PM_2.5_. The total races of the conditional group showed lower Rho values and significant associations of the same trend for CO, NO_2_, NO and SO_2_. Conclusions: Horse races between 1000 and 1200 m of the “Handicap” type appear to be an interesting group to study the relationship between air pollutants and the performance of racehorses. In the future, our observations should be expanded to other distances and other types of races.

## 1. Introduction

Physical performance is an area of broad interest in both animal and human sport competitions. The factors affecting this variable are numerous. There are performer’s own, or intrinsic, factors, such as genetic load [1], degree of training [2], biomechanical aspects, energy cost of movements [3], type of metabolism used, magnitude of maximal oxygen consumption [4], or age [5]. Psychological factors, race strategy and behavior are also intrinsic variables [6]. Another large group of variables, classified as extrinsic, correspond to the conditions in which the competition takes place, such as the distance of the race, the type, condition and design of the track, wind speed, humidity, ambient temperature, or geographical altitude. Ambient air pollutants (please see details in the technical report by the WHO [7]) also belong to this group, which, from a theoretical point of view, are widely recognized as potentially influencing performance; but the magnitude of their influence has been little described. From the ambient air point of view, the problem is constituted both by the great variability of the mixture and by the fact that its components affect performance by more than one mechanism. Thus, in the case of carbon monoxide (CO), for example, this gas binds to blood forming carboxyhemoglobin, which affects oxygen transport [8], while particulate matter (PM_10_ and PM_2.5_), nitrogen oxides (NO_2_ and NO), sulfur oxides (SO_3_ and SO_2_) and ozone (O_3_) act as irritants and inflame the airway, favoring the formation of secretions and increasing their resistance [9,10]. In view of the complexity of the problem, this question has been rarely addressed, and when it has been addressed, it referred to the laboratory application of individual contaminants in humans [11,12]. There are also a limited number of studies that have used values obtained in competitions, some of them made up of groups of human athletes of very different levels of performance [13]. To our knowledge, the only study on horse racing assessed the effect of air pollution on performance in 675 Grade I Stakes Thoroughbred racing conducted over 35 years at various US racetracks, for distances ranging between seven and twelve furlongs (about 1400 and 2400 m, respectively). The author described a marked decrease in the level of performance at high concentrations of O_3_ (unusual) with an overall minimal effect of the pollutants under the studied environmental conditions [14].

Experimental implementation to study the relationship between air pollutants and performance, under ideal conditions, would involve having a group of athletes from a similar gene pool and with comparable training and nutrition, as well as similar baseline environmental exposure, performing at their best in multiple tests in the presence of air with great variability in composition; which clearly represents a challenge of great magnitude. In this way, the study of a natural experiment, such as horse racing, may be an interesting approach, because these competitions are carried out by thoroughbred race horses; a group of animal athletes, bred, selected and trained specifically for these purposes that compete in multiple opportunities during the year in many countries around the world [15]. In addition, horse racing is highly regulated, which gives rise to varied types of races, designs and track surfaces that can potentially affect the model. For this reason, it is necessary to establish the best conditions to generate an adequate corpus of study to analyze the problem. Thus, in this paper we have created a dataset of official thoroughbred horse races in which we have applied some theoretical principles to establish a study model for the relationship between air pollutants and performance. Once these theoretical principles were applied, we compared a group of races of relative homogeneity, in terms of race conditions and competitors, versus a group of more heterogeneous races to evaluate our selection method.

## 2. Materials and Methods

**Figure 1:**
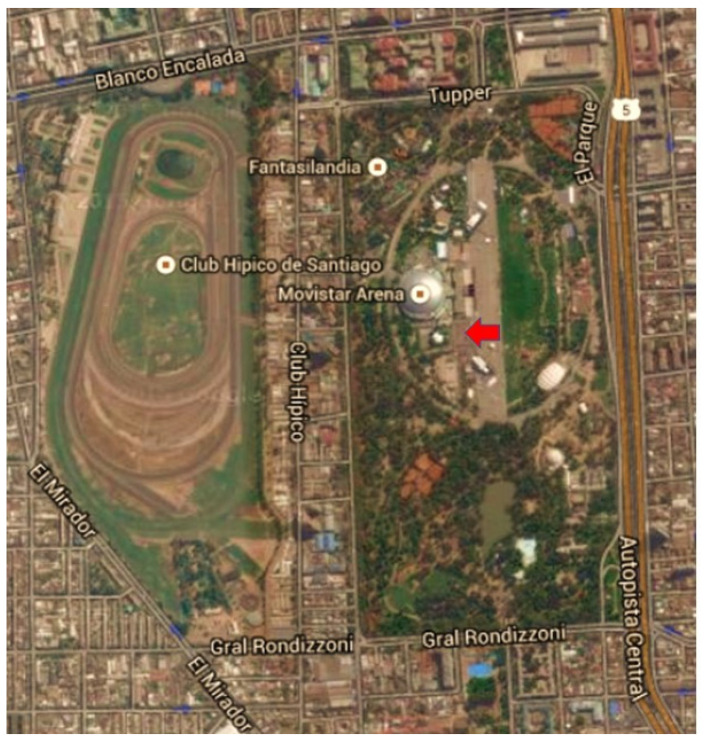
Satellite map of the “Club Hípico de Santiago”. The monitoring station “Parque O’Higgins” is indicated in red. The image was obtained from the Google maps site https://www.google.cl/maps (accessed on 13 March 2022).

Air pollutants: pollutant concentrations were obtained 470 m from the equestrian center at the “Parque O’Higgins” station (see Figure 1), which is part of the air quality monitoring network of the city of Santiago, Chile (http://macam.mma.gob.cl (accessed on 1 February 2022). According to information from the environmental network, NO_2_, NO and O_3_ gases (expressed in ppm) were determined by chemiluminescence. For SO_2_, ultraviolet fluorescence was used, while CO concentration (expressed in ppm) was determined by dispersive infrared photometry. Finally, the determination of particulate matter (PM_10_ and PM_2.5_, (expressed in µg/m^3^) was carried out by gravimetry. The time of the winning horse, names and sexes of the horses, as well as the schedule, type of race, distance, track design and condition, were obtained from the official website of the “Club Hípico de Santiago” equestrian center (http://www.clubhipico.cl/ (accessed on 1 February 2022)). Since the data was in the public domain, no ethical evaluation was required.

Dataset: The sample included 441 official races carried out on an open 2000-m-long, 25-m-wide sand or grass track. The selected races were carried out over a total period of eight months during the summer (January, February) and winter (May, June, July, and August) of 2012. The races ranged from 1000 to 2000 m in length with varying layouts according to the distance, and the track conditions were described by the organizers as “good”, “regular” or “heavy”. The “Classics” type races (n = 24) were eliminated from our analysis because their competitors were selected for very high previous performance. Thus, the races analyzed were classified as “Handicap”, in which thoroughbred racehorses of both sexes competed, grouped according to a similar level of performance established by a ranking, to which an additional weight was added to balance the chances of winning the race. The other category was for “Conditional” races that included some mechanism for selecting participants according to their previous results, by being categorized as “winners”, “losers”, and those in which animals ran separated by sexes (e.g., “winning females”, “losing males”) or by age (e.g., “winning three-year-old males”).

Analysis strategy: according to the seasonal variation profile of pollutants typical of the city of Santiago, Chile, and with the purpose of generating the greatest variability in the composition of the ambient air, we included races carried out in summer and winter months. In addition, knowing that there can be immediate and delayed effects of pollutants on the animals, it was strategically chosen to measure the “delayed effect” by using the mean of the pollutants in the six hours prior to the competitions for the subsequent analysis. In order to reduce variation in performance, due to extrinsic and intrinsic factors, only the winning animal’s time was included for races carried out on a similar track (one curve and one straight) at short distances (1000, 1100 and 1200 m) to reduce the effect of strategy, and with a track described by the organizers as “good”. The tests studied were carried out in a short period of time (months), on a single racetrack to include a limited number of observed animals observed. Regarding the types of races carried out at the racetrack, these are highly standardized and regulated by the organizers. In this regard, to participate in the “Handicap” races, the horses must achieve a good level of performance. Based on these sporting achievements, they are ranked, assigned a “handicap index” and grouped according to this ranking when competing. In addition, within each race, they are assigned weights according to their ranking, which, according to the organizers, is intended to equalize the possibility of winning the race. From a theoretical point of view, this group appears as an opportunity to study our problem, since it has a relative homogeneity in terms of its participants and the conditions of competition. To evaluate the usefulness of this group, we compared it versus the “Conditional” races where there are different criteria to compete, making it more heterogeneous. For this comparison, correlations were calculated between race speed and pollutant concentration at the distances described. In addition, to give strength to our observations, the correlations were performed more than once, when studied at each distance and group, and were calculated after grouping the three distances to give a truer view of the associations and increase the statistical power of the associations.

Statistical analysis: in the “Handicap” and “Conditional” race groups, the basic statistics were obtained, and the type of distribution was determined by applying the D’Agostino and Pearson test for each variable. To describe any differences and compare the pollutant levels between the distances analyzed, according to the type of distribution, an ANOVA or Kruskal-Wallis test was performed, using Tukey’s or Dunn’s test as a posteriori test, respectively. When differences were detected, the “Eta squared” was calculated to evaluate the size of the difference. Subsequently, the data of the three distances were collected, for which a similar analysis was performed. Depending on the group distribution, the unpaired t-test or the Mann-Whitney test was used, and then, if differences were detected, Cohen’s d was calculated. The magnitude of the differences observed in both cases were interpreted according to Cohen’s criteria [16]. Finally, the correlations between race speed and individual pollutant concentrations were obtained using Spearman’s method; a significant value being considered when p was less than 0.05. Statistical analysis was performed using GraphPad Prism (version 9.0; San Diego, CA, USA).

## 3. Results

**Figure 2:**
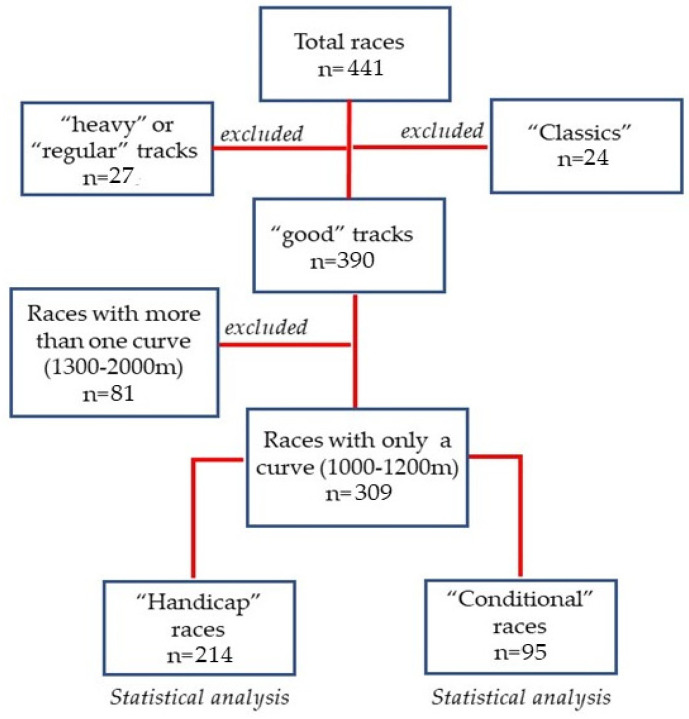
Data analysis inclusion and exclusion criteria. First, a “Classic” type race with a “heavy” or “regular” track was excluded. Then, those that included more than one curve were excluded by track design. Finally, statistical analysis was performed in the 1000, 1100 and 1200 m races comparing the “Handicap” races with the “Conditional” races.

Of the 441 total races, using our analysis strategy, 27 (6.12%) were discarded because the track was described as “heavy” or “regular”, and then 81 (18.36%) because of their racetrack morphology (see Figure 2). Thus, the two groups where the effect of the homogeneity of the races on the pollutants, versus performance, was compared consisted of 214 (48.5%) races of the “Handicap” type and 95 (21.54%) of the “Conditional” type. Table S1 summarizes the mean, standard deviation, and interquartile range for the run speed and concentration of pollutants in the “Handicap” races. There, a lower concentration of PM_2.5_ can be observed in the 1100-m races compared to the 1000-m races, with a small difference in size. The same parameters for the “Conditional” races are presented in Table S2, which shows a lower concentration of PM_2.5_, CO, NO_2_ and NO at 1100 m versus 1200 m. This same difference is seen for PM_10_, in addition to a lower value for the 1000-m races. With respect to O_3_, the trend is reversed, with a higher concentration at 1100 versus 1200 m. Regarding the size of the difference, the pollutants that presented changes showed a medium value. It was also evaluated whether the load of the different pollutants was similar for the total of the “Handicap” versus “Conditional” races. In this regard, it was observed that the group of “Conditional” races was exposed to a higher concentration of PM_2.5_, CO, NO and SO_2_ with the magnitude of the difference observed being small (see Table 1). To evaluate the heterogeneity of the “Conditional” group, we compared the determinants of race speed, according to the various subtypes of tests of this category (see methods section); thus, we performed a 2 × 2 ANOVA test, using the sex of the animals and their previous results (winners/non-winners) as factors considered in selecting the competitors for these races. The factor “previous results” proved to be the determinant (p = 0.0007), the factor “animal sex” did not appear to be a determinant of the result (p = 0.12), while the interaction between the factors was significant (0.041). The analysis by group showed that the winning females (n = 9; 63.43 km/hr) were faster than the non-winning females (n = 37; 62.54 km/hr), value of p = <0.0001, with a large difference size (d = 1.81). Regarding the group of males, no differences were observed. Since neither of these two factors were used to select the competitors in the “Handicap” group, it was not possible to make a similar comparison.

Table 2 shows the Spearman’s Rho calculated for the correlations studied, and the *p*-values for the associations between speed and pollutant concentration in the “Handicap” group. In these tables, it is possible to observe that, at 1000 m, there are negative correlations with PM_10_, PM_2.5_, CO, NO_2_, NO and SO_2_, while with O_3_ the correlation is positive. In the 1100-m group, the same trends are maintained, but with lower Rho values, except for CO. At 1200 m, the same trends can be seen, with lower Rho values for CO, NO2, NO and SO2, without significant associations.

Regarding the “Conditional” races, Table 3 shows the Spearman’s Rho values and the *p*-values calculated for the speed/pollutant correlations. The 1000-m races show correlations with the same trends as those found in the “Handicap” group, except for PM_10_ and PM_2.5_, but with lower Rho values for NO_2_ and SO_2_. In turn, in this group higher CO and O_3_ values were found with respect to the first group. At 1100 m, lower Rho values than in the first group analyzed for this type of race were observed, and only CO and SO_2_ showed a significant association. Also, when comparing their values with the same distance as the “Handicap” group, lower Rho values were observed in this group of races. In the case of 1200 m, we did not find significant associations. Finally, when analyzing the correlations with the total data of each category, the “Handicap” group presented significant associations of speed with all pollutants, with the same trend observed in each distance of this group, while the group of “Conditional” races showed lower Rho values than the “Handicap” group for all pollutants and presented significant correlations only for CO, NO_2_, NO and SO_2_. It should be noted that, in view of the multiple comparisons made between speed and pollutants, the possibility of making the type I error increases. Using the *p*-value adjustment criterion with the use of the Bonferroni adjustment for the total dataset of each category, we found that the described significances are maintained for all the correlations of the “Handicap” group, while the significance is only maintained in the relationship between the winning horse’s speed and SO_2_.

## 4. Discussion

In this paper, we developed a dataset and applied theoretical principles for its analysis in search of a study model for the relationship between air pollutants and performance in racehorses. Our analysis showed that race categories may add unwanted variability to an eventual model, so any tendency to correlate data for all races and conditions involved in horse racing must be treated with caution. For this reason, one of the first tasks was to look for the appropriate group of races for our purposes. Thus, in view of wanting to establish the most standardized conditions, in terms of design and track conditions (see Figure 2), we considered that the “Handicap” type of races might constitute a corpus of analysis. This is because to take part in this type of race, animals of both sexes must comply with age requirements and sporting achievements. In addition, at the time of the race, an attempt is made to equalize the conditions of success among the participants. In this way, the “Handicap” type of races generates a degree of homogeneity that transforms these races into a group of interest to solve our research problem. Thus, as a way of evaluating the effect of the homogeneity of these races on the relationship under study, we compared this group against a group of races of great heterogeneity, where performance is strongly influenced by the selection of the sample.

The first step of this analysis consisted in characterizing the pollutant concentrations to which the animals were exposed, which can be seen in Tables S1 and S2. Using the medians of the total races presented in Table 1 as a parameter, we found that the concentrations at which these races were conducted are above the suggested pollutant exposure norms for humans according to WHO [17]. Thus, this institution recommends for PM_10_ an annual mean of 10 versus the 84–88 µg/m^3^ found in our races. For PM_2.5_, an annual mean of 5 is recommended, versus a range between 22–23 µg/m_3_ present in our sample. For NO_2_ an annual mean of 5.14 is recommended versus the observed value between 16–21 ppb, or a daily maximum value of 29.56, taken as an 8 h mean over 6 months is recommended, while in our sample observed values varied between 27 to 32 ppb. This is relevant, since our observations were made in a highly contaminated area. The explanation for this has to do with the fact that the city of Santiago, Chile, has high general levels of pollution [18], which is exacerbated by the fact that the track analyzed is located about 800 m from a large interprovincial highway that has a high vehicular flow (see “Autopista Central” in Figure 1). The fact that it is an area with high levels of pollution allowed us to sample animals exposed to multiple mixtures of pollutants that occur naturally in the environment. Exposing animals to such multiple levels of pollutants in a laboratory would be difficult to reproduce, due to ethical concerns when proposing such tests in view of the known effects of the pollutants. From another aspect, it is important to remember that these animals are bred and live in environments with high pollution within their stables with the presence of dust and endotoxins [19], which has been linked to recurrent coughing and inflammatory airway disease in horses, [20] which, in turn, is linked to poor athletic performance. In our case, the horses grew up and trained in an environment with high levels of other pollutants, which may influence their acute response to exercise in these conditions [21,22]. This should be studied in the future against data obtained from racetracks with better air conditions.

The study of the association between speed and pollutants is presented in Table 2. Strategically, we made the comparison by distance in both groups and once we found that the speed of the race did not vary in both cases, we were able to gather the data from these races, which allowed us to repeat the correlations more than once. In the “Handicap” group, the Rho values tended to be lower as the distance increased, and we found significant associations for all pollutants at 1000 and 1100 m but not at 1200 m. A general comparison with the “Conditional” group showed that, in this group, for the most part, there were lower Rho values for the same distance, with the most significant associations occurring at the shortest distance. In another aspect, regarding the potential effect on correlations of different pollutant loads in the “Conditional” group, there is no difference in pollutants between 1000 and 1100 m (see Table S2). Regarding the 1200 m results, given that the weakest associations (lower Rho values) were seen at this distance, we speculate that this is largely a reflection of the application of race strategies, but this will need to be resolved in other studies. In any case, it is worth mentioning that, for the “Handicap” group, the Rho values obtained for this distance are lower, but with the same trend as those of the previous distances, so that the absence of significance may be explained by a low number of races at this distance. Additionally, this is reinforced because, when calculating the Rho for the three distances, significant inverse relationships appear that respond to a biological logic in view of the effects that pollutants can generate in exercising animals, such as increased resistance and inflammation of the airway [23]. This was true for most of the pollutants, except for O_3_, the gas with the lowest Rho value. Speculatively, perhaps its performance-lowering action is more acute due to its irritant effect on the airway [24]. However, it should be noted that the minimum concentration of the gas that induces a bronchoconstrictor effect in exercise has been described in humans at concentrations five times higher than those reported here [25]. Alternatively, it is possible that the association found reflects its relationship with other factors, such as environmental temperature (not currently studied), in view of the fact that its concentration increases in the warmer and brighter months [26].

Regarding the limitations of our research, there was a low percentage of races in which horses who regularly competed in the “Handicap” races also competed in the “Conditional” races. We believe that the effect of this overlapping of competitors is lessened because we only considered the winning horse of the races, but, even so, 14 horses won races in both types of races, which represented 5% of the total of both groups. Another limitation inherent to the nature of the study is that, regardless of the correlations described and the biological reasoning of our findings, we cannot affirm that the correlations are cause and effect, and we lack a complete study of the phenomenon to do so. Finally, at the single racetrack analyzed, most of the programmed races are carried out at distances between 1000 and 1200 m; however, a large number of races are held over longer distances.

The observed decreased level of performance at high concentrations of pollutants, is associated with the fact that exercise causes ventilation to increase. This phenomenon in humans is associated with an increase in the deposition of particulate material in the lungs [27]. In addition, when O_3_ is present, chest pain, dyspnea and wheezing appear [28] and, in some cases, airway reactivity increases, expressed in a lower FEV_1_ [29]. A similar result has been reported for the administration of particulate matter [30]) and SO_2_ [31]. In humans, exercise produces inflammation and oxidative lung damage [32,33]). In the same direction, Ivester et al. in 2018 found a high percentage (80%) of inflammatory patterns, compatible with moderate asthma, in 64 racehorses when analyzing bronchial-alveolar lavage. This process was associated with increased exposure to particulate matter below 4 μm and lower performance of the animals [34]. The phenomena described above may affect the level of performance by limiting O_2_ intake, increasing the work of breathing [35] and may eventually affect the exchange of O_2_ and CO_2_ at tissue level. From the cardiovascular point of view, during exercise, particulate matter has been associated with increased vascular reactivity, due to increased sympathetic activity [36,37]. In addition, in humans, a decrease in physical performance was reported from exposure to high concentrations of CO in comparison with our data (100 ppm), affecting both oxygen transport and evidencing cardiac malfunction [38]. Thus, it is possible that the decreased level of performance may be, in part, associated with a deficit in delivery due to a deficit in cardiovascular action (cardiac pump/blood vessels).

Regarding the projections of the presented study presented, it represents in itself a contribution to horseracing as a whole (breeders, owners, organizers). Furthermore, it is relevant to conduct this study on horses, given the eventual translational implications for humans, since several studies pn horses have been used as a model in the search for mechanisms in areas such as exercise physiology [39,40], diseases of the musculoskeletal system, such as osteoarthritis [41] and tendon damage [42], as well as in the study of tissue regeneration [43,44] and immunological processes linked to exercise [45].

## 5. Conclusions

Thoroughbred racehorses are a highly selected and trained group that compete in many parts of the world, in diverse environmental conditions. In view of these conditions, this activity appears an opportunity to advance the understanding of the effect of air pollution on physical performance. In addition, it is possible that within a standardized and diverse activity there are types of races that can be a good model for study. Thus, according to our results, we believe that horse racing (<1200 m) in the “Handicap” category can constitute a good group to study the relationship between pollutants and performance.

## Figures and Tables

**Table 1:** Comparison between the race speed and pollutant concentration of all races in the “Handicap” versus “Conditional” groups. The *p* value shown corresponds to the t-student or Mann-Whitney test, according to the distribution of the data, in addition to Cohen’s “d”, calculated when there were differences. Values are expressed as mean ± standard deviation and median (IQR).

	Handicap	Conditional	*p*-Value/
			Cohen’s d
**Races (n)**	**214**	**95**	
Speed (km/h)	62.36 ± 0.89	62.25 ± 0.91	0.32
	62.43 (1.22)	62.19 (1.37)	
PM_10_ (µg/m^3^)	90.42 ± 31.61	92.25 ± 31.04	
	86.50 (44.25)	84.30 (37.00)	0.94
PM_2.5_ (µg/m^3^)	24.97 ± 12.47	29.25 ± 15.10	
	21.35 (16.05)	23.90 (18.10)	0.0247/0.25
Ozone (ppb)	25.66 ± 12.69	28.68 ± 13.23	
	27.80 (22.28)	31.70 (23.60)	0.086
CO (ppm)	0.44 ± 0.29	0.56 ± 0.44	
	0.35 (0.40)	0.30 (0.40)	0.033/0.23
NO_2_ (ppb)	26.15 ± 21.33	28.61 ± 24.46	
	17.60 (37.75)	16.50 (28.10)	0.14
NO (ppb)	16.12 ± 28.07	33.34 ± 46.64	
	4.65 (18.88)	10.90 (35.50)	<0.0001/0.48
SO_2_ (ppb)	2.73 ± 1.25	3.17 ± 1.56	
	2.30 (1.12)	2.60 (2.00)	0.044/0.23

**Table 2:** Spearman’s Rho (above) and *p*-values (in italics below) calculated between the winning horse’s speed and the mean values of the pollutant concentration during the six hours prior to the race in the “Handicap” category for the distances of 1000, 1100, 1200 m and for all the races together. The n for O_3_ were 32, 138, 30 respectively, while for the total races it was 200. Significant *p*-values using the Bonferroni adjustment are shown in bold italics.

	1000 m	1100 m	1200 m	Total
Races (n)	33	148	32	214
PM_10_	−0.65 ***0.000037***	−0.25 ***0.0019***	−0.25 *0.16*	−0.32 ***0.0000015***
PM_2.5_	−0.63 ***0.000060***	−0.25 ***0.0019***	−0.34 *0.059*	−0.31 ***0.0000026***
Ozone	0.44 *0.012*	019 *0.024*	0.20 *0.28*	0.20 ***0.0038***
CO	−0.42 *0.014*	−0.39 ***0.0000010***	−0.26 *0.16*	−0.37 ***0.000000018***
NO_2_	−0.64 ***0.000048***	−0.35 ***0.000011***	−0.30 *0.099*	−0.40 ***0.0000000021***
NO	−0.58 ***0.00034***	−0.38 ***0.0000019***	−0.18 *0.32*	−0.38 ***0.0000000096***
SO_2_	−0.67 ***0.000013***	−0.27 ***0.00082***	−0.18 *0.32*	−0.33 ***0.00000061***

**Table 3:** Spearman’s Rho (above) and *p*-values (in italics below) calculated between the winning horse’s speed and the mean values of the pollutant concentration during the six hours prior to the race in the “Conditional” category for the distances of 1000, 1100, 1200 m and for all the races together. The n for O_3_ were 17, 51, 19 respectively, while for the total races it was 87. Significant *p*-values, using the Bonferroni adjustment. are shown in bold italics.

	1000 m	1100 m	1200 m	Total
Races (n)	17	55	23	95
PM_10_	0.045 *0.86*	−0.15 *0.27*	−0.21 *0.34*	−0.04 *0.67*
PM_2.5_	−0.46 *0.063*	−0.07 *0.63*	−0.25 *0.24*	−0.16 *0.13*
Ozone	0.48 *0.050*	−0.05 *0.73*	0.07 *0.76*	0.03 *0.79*
CO	−0.59 *0.015*	−0.28 *0.038*	−0.06 *0.78*	−0.23 *0.023*
NO_2_	−0.42 *0.093*	−0.22 *0.10*	−0.17 *0.44*	−0.26 *0.010*
NO	−0.56 *0.020*	−0.19 *0.18*	−0.23 *0.30*	−0.23 *0.024*
SO_2_	−0.62 *0.010*	−0.35 *0.010*	−0.17 *0.43*	−0.32 ***0.0015***

## Data Availability

The database is included in the Supplementary Materials.

## Supplementary Materials

The following supporting information can be downloaded at: https://www.mdpi.com/article/10.3390/ani12091139/s1, Table S1: Comparison of air pollutant load and speed of the “Handicap” races. Table S2: Comparison of air pollutant load and speed of the “Conditional” races. Table S3: Dataset of “Handicap” races; Table S4: Dataset of “Conditional” races.
