# Supplementary material for: Horse Racing as a Model to Study the Relationship between Air Pollutants and Physical Performance
Source: Animals (Basel). 2022 Apr 28;12(9):1139. doi: 10.3390/ani12091139 (PMC9103629; doi:10.3390/ani12091139)
Supplement: Supplementary file 1 [file animals-12-01139-s001.zip › animals-1663588-supplementary.pdf]

Supplementary Materials

Table S1. Race speed of the 1,000; 1,100 and 1,200 meter races. In addition, the mean concentrations of pollutants measured in the ambient air six hours before the start of the "Handicap" type races are shown. The p value shown corresponds to the ANOVA or Kruskal-Wallis test, respectively. The values are expressed as mean±standard deviation and as median (IQR). The n for O_3 were 31, 138, 30 respectively, while for the total races it was 169. "a" is different from the 1,000 meter group.

	**1,000 m**	**1,100 m**	**1,200 m**	**p-value/ Eta squared**
**Races (n/%)**	34/15.88	148/69.16	32/14.95	
**Speed (km/h)**	62.05±0.90	62.42±0.94	62.41±0.55	0.09
	61.95 (1.56)	62.47(1.23)	62.50(0.98)	
**PM_{10} (μg/m^3)**	99.22±37.91	88.44±28.83	90.18±35.89	0.20
	88.25(57.22)	87.50(45.21)	82.58(51.21)	
**$PM_{2.5}$ (μg/m^3)**	29.33±12.44	23.73±11.67	26.06±15.12	
	23.90(20.90)	21.32(16.00)[a]	20.71(7.96)	0.03/0.024
**Ozone (ppb)**	26.47±11.22	24.73±13.40	29.09±10.26	
	29.00(20.53)	27.56(24.43)	32.43(16.03)	0.17
**CO (ppm)**	0.50±0.38	0.44±0.27	0.40±0.28	
	0.40(0.50)	0.36(0.40)	0.24(0.26)	0.08
**NO_2 (ppb)**	30.11±22.09	26.04±20.80	22.44±22.88	
	29.00(40.43)	18.52(37.92)	8.86(28.96)	0.49
**NO (ppb)**	22.32±43.07	14.96±23.05	14.91±29.52	
	7.40(18.20)	5.14(19.31)	2.28(16.11)	0.58
**SO_2 (ppb)**	3.20±1.81	2.58±0.85	2.92±1.86	
	2.95(2.60)	2.39(1.02)	2.06(1.24)	0.64

Table S2. Race speed of the 1,000; 1,100 and 1,200 meter races. In addition, the mean concentrations of pollutants measured in the ambient air six hours before the start of the "Conditional" type races are shown. The p-value shown corresponds to the ANOVA or Kruskal-Wallis test respectively. Values are expressed as mean±standard deviation and median (IQR). The n for O_3 were 19, 51, 23 respectively, while for the total races it was 93. "c" is different from the 1,200 meter group.

	**1,000 m**	**1,100 m**	**1,200 m**	**p-value/ Eta squared**
**Races (n/%)**	**17/17.89**	**55/57.89**	23/24.21	
**Speed (km/h)**	62.27±1.33<br>61.62(2.41)	62.15±0.84<br>62.2(1.36)	62.48±0.64<br>62.39(0.89)	0.34
**PM_{10} (μg/m^3)**	82.25±26.14<br>73.80(24.25)^C	86.58±26.88<br>84.00(28.33)^C	113.20±36.4<br>114.30(67.17)	0.0036/0.10
**$PM_{2.5}$ (μg/m^3)**	29.01±13.09<br>25.30(14.45)	24.56±11.48<br>21.58(12.17)^C	40.61±18.28<br>44.50(34.84)	0.0008/0.13
**Ozone (pub)**	25.42±13.59<br>27.70(24.55)	32.4±12.25<br>34.68(13.88)^C	21.62±12.32<br>21.47(22.24)	0.0063/0.092
**CO (ppm)**	0.63±0.35<br>0.70(0.50)	0.42±0.27<br>0.31(0.21)^C	0.87±0.63<br>0.70(1.23)	0.038/0.097
**NO_2 (ppb)**	31.41±23.16<br>35.30(30.80)	21.32±17.88<br>13.01(19.36)^C	43.97±31.53<br>37.2(64.95)	0.010/0.076
**NO (ppb)**	26.35±30.05<br>17.00(33.90)	21.05±35.17<br>4.87(24.49)^C	67.86±62.77<br>54.54(106.34)	0.0041/0.098
**SO_2 (ppb)**	3.10±0.95<br>34.00(1.80)	2.84±1.36<br>2.44(1.16)	4.04±2.06<br>4.31(3.26)	0.086

Table S3. Dataset of handicap races.

Number	Distance	Type of race	Track Condition	Speed	PM10	PM2.5	O3	CO	NO2	NO	SO2
173	1000	Handicap	Good	63.62	70.9	18.4	39.6	0.2	6.0	1.0	2.1
182	1000	Handicap	Good	61.46	64.4	26.7	21.0	0.4	17.8	28.1	1.8
191	1000	Handicap	Good	62.60	58.3	21.2	37.3	0.2	7.5	1.8	1.4
193	1000	Handicap	Good	62.98	59.9	17.8	36.7	0.2	6.8	1.5	1.4
194	1000	Handicap	Good	62.86	59.9	17.8	36.7	0.2	6.8	1.5	1.4
195	1000	Handicap	Good	61.89	56.0	17.9	34.0	0.2	6.4	1.3	1.5
196	1000	Handicap	Good	63.21	56.0	17.9	34.0	0.2	6.4	1.3	1.5
197	1000	Handicap	Good	62.38	55.8	17.8	29.0	0.2	7.0	1.2	1.6
198	1000	Handicap	Good	63.06	55.8	17.8	29.0	0.2	7.0	1.2	1.6
201	1000	Handicap	Good	62.26	97.8	32.3	17.2	0.6	27.8	38.1	1.9
212	1000	Handicap	Good	62.60	88.7	19.3	35.2	0.3	8.1	1.2	2.0
213	1000	Handicap	Good	64.00	91.3	19.7	34.3	0.3	8.3	1.0	2.0
214	1000	Handicap	Good	61.96	91.3	19.7	34.3	0.3	8.3	1.0	2.0
216	1000	Handicap	Good	62.84	93.6	20.0	31.7	0.3	8.7	1.0	2.0
217	1000	Handicap	Good	62.37	86.5	19.9	28.9	0.3	8.9	1.0	2.0
237	1000	Handicap	Good	61.38	165.4	59.9	8.4	2.0	81.9	178.1	7.7
249	1000	Handicap	Good	61.92	134.7	41.7	40.1	0.7	62.7	7.4	5.0
250	1000	Handicap	Good	61.29	134.7	41.7	40.1	0.7	62.7	7.4	5.0
252	1000	Handicap	Good	61.59	152.3	40.2	34.9	0.7	57.7	6.7	4.7
253	1000	Handicap	Good	61.07	152.3	40.2	34.9	0.7	57.7	6.7	4.7
254	1000	Handicap	Good	61.30	183.1	42.8	26.8	0.8	57.5	8.9	4.4
255	1000	Handicap	Good	60.95	183.1	42.8	26.8	0.8	57.5	8.9	4.4
266	1000	Handicap	Good	62.41	74.3	28.9	11.1	0.7	45.2	16.6	2.9

293	1000	Handicap	Good	61.00	123.8	49.6	4.7	1.5	54.0	178.6	5.3
301	1000	Handicap	Good	61.94	86.5	27.1	15.5	0.5	46.5	33.3	3.3
305	1000	Handicap	Good	62.24	87.8	21.4	14.3	0.4	37.7	11.5	3.0
306	1000	Handicap	Good	60.93	87.8	21.4	14.3	0.4	37.7	11.5	3.0
307	1000	Handicap	Good	61.20	87.8	21.4	14.3	0.4	37.7	11.5	3.0
308	1000	Handicap	Good	60.93	108.8	23.9	11.6	0.4	37.3	12.0	3.0
309	1000	Handicap	Good	61.30	108.8	23.9	11.6	0.4	37.3	12.0	3.0
322	1000	Handicap	Good	62.82	84.3	29.7	42.6	0.5	30.4	3.9	3.2
329	1000	Handicap	Good	60.60	106.0	41.4		0.1	22.4	39.1	6.6
339	1000	Handicap	Good	61.13	154.8	60.5		0.6	29.8	81.1	8.2
577	1000	Handicap	Good	63.58	71.1	34.5	16.0	0.9	28.2	41.6	2.2
140	1100	Handicap	Good	61.04	102.0	15.5	41.1	0.2	6.0	1.0	2.1
1	1100	Handicap	Good	63.01	89.8	31.4	27.4	0.4	18.9	14.8	2.4
10	1100	Handicap	Good	63.15	74.7	22.3	45.4	0.2	6.3	1.0	2.6
11	1100	Handicap	Good	64.83	76.9	20.6	44.8	0.2	6.4	1.0	2.8
14	1100	Handicap	Good	62.88	80.0	20.3	42.5	0.2	6.9	1.0	2.8
15	1100	Handicap	Good	63.06	79.3	20.3	38.6	0.2	7.8	1.0	2.7
16	1100	Handicap	Good	63.19	79.3	20.3	38.6	0.2	7.8	1.0	2.7
17	1100	Handicap	Good	62.93	79.3	20.3	38.6	0.2	7.8	1.0	2.7
18	1100	Handicap	Good	61.85	43.7	13.6	27.3	0.3	7.2	2.4	1.7
24	1100	Handicap	Good	62.82	64.8	12.1	34.8	0.3	6.0	1.3	1.8
29	1100	Handicap	Good	63.33	95.4	11.8	32.6	0.3	5.4	1.1	1.9
30	1100	Handicap	Good	62.32	109.2	12.1	28.9	0.2	5.5	1.2	1.9
31	1100	Handicap	Good	62.35	109.2	12.1	28.9	0.2	5.5	1.2	1.9
32	1100	Handicap	Good	62.88	119.8	12.7	24.9	0.2	6.0	1.4	1.9
33	1100	Handicap	Good	63.21	119.8	12.7	24.9	0.2	6.0	1.4	1.9
34	1100	Handicap	Good	62.71	119.8	12.7	24.9	0.2	6.0	1.4	1.9
35	1100	Handicap	Good	63.41	119.5	12.4	21.2	0.3	6.9	1.5	1.9

36	1100	Handicap	Good	62.18	85.3	24.3	29.6	0.4	17.4	11.3	2.1
47	1100	Handicap	Good	63.07	104.4	24.3	47.0	0.3	8.3	1.1	1.9
48	1100	Handicap	Good	63.61	104.2	24.1	43.6	0.3	8.1	1.0	2.0
50	1100	Handicap	Good	62.76	102.2	24.2	39.0	0.3	8.3	1.0	2.0
51	1100	Handicap	Good	62.43	102.2	24.2	39.0	0.3	8.3	1.0	2.0
52	1100	Handicap	Good	62.59	102.2	24.2	39.0	0.3	8.3	1.0	2.0
53	1100	Handicap	Good	63.32	94.6	23.2	34.7	0.3	8.7	1.0	1.9
54	1100	Handicap	Good	63.53	94.6	23.2	34.7	0.3	8.7	1.0	1.9
55	1100	Handicap	Good	62.09	65.7	16.9	18.2	0.2	10.2	7.0	2.0
65	1100	Handicap	Good	63.73	62.5	14.7	31.1	0.2	5.4	1.0	2.1
67	1100	Handicap	Good	62.08	62.9	16.0	30.1	0.2	5.8	1.0	2.2
68	1100	Handicap	Good	63.42	62.9	16.0	30.1	0.2	5.8	1.0	2.2
69	1100	Handicap	Good	62.57	62.9	16.0	30.1	0.2	5.8	1.0	2.2
70	1100	Handicap	Good	63.16	62.3	16.6	27.7	0.2	6.5	1.0	2.2
71	1100	Handicap	Good	62.95	62.3	16.6	27.7	0.2	6.5	1.0	2.2
72	1100	Handicap	Good	63.54	61.3	16.6	24.3	0.2	7.3	1.0	2.2
91	1100	Handicap	Good	62.02	96.5	23.3	24.9	0.5	19.9	20.6	3.3
101	1100	Handicap	Good	62.11	81.8	15.8	39.8	0.2	6.7	1.0	2.6
103	1100	Handicap	Good	64.16	82.5	15.4	37.5	0.2	6.7	1.0	2.5
104	1100	Handicap	Good	63.55	82.5	15.4	37.5	0.2	6.7	1.0	2.5
105	1100	Handicap	Good	63.81	82.7	16.3	34.3	0.2	7.0	1.0	2.4
106	1100	Handicap	Good	62.79	82.7	16.3	34.3	0.2	7.0	1.0	2.4
107	1100	Handicap	Good	62.42	82.7	16.3	34.3	0.2	7.0	1.0	2.4
108	1100	Handicap	Good	62.43	76.1	14.8	30.6	0.2	7.3	1.0	2.3
109	1100	Handicap	Good	63.46	76.1	14.8	30.6	0.2	7.3	1.0	2.3
110	1100	Handicap	Good	61.53	62.1	17.6	21.6	0.4	15.7	18.7	2.1
121	1100	Handicap	Good	63.50	54.5	9.9	35.1	0.2	5.5	1.0	2.0
122	1100	Handicap	Good	62.97	53.8	9.0	32.0	0.2	5.4	1.0	2.0

123	1100	Handicap	Good	63.28	53.8	9.0	32.0	0.2	5.4	1.0	2.0
124	1100	Handicap	Good	62.88	51.5	8.0	27.8	0.2	5.6	1.0	2.0
125	1100	Handicap	Good	63.71	51.5	8.0	27.8	0.2	5.6	1.0	2.0
126	1100	Handicap	Good	62.39	51.5	8.0	27.8	0.2	5.6	1.0	2.0
127	1100	Handicap	Good	62.93	47.9	7.7	23.6	0.2	6.0	1.0	2.0
128	1100	Handicap	Good	60.86	99.1	26.1	27.3	0.7	33.9	40.1	3.3
138	1100	Handicap	Good	63.09	98.0	15.6	44.4	0.2	6.2	1.0	2.2
139	1100	Handicap	Good	63.49	98.0	15.6	44.4	0.2	6.2	1.0	2.2
141	1100	Handicap	Good	61.99	102.0	15.5	41.1	0.2	6.0	1.0	2.1
143	1100	Handicap	Good	61.87	114.2	20.2	38.1	0.2	6.4	1.0	2.0
144	1100	Handicap	Good	61.42	119.3	24.4	35.9	0.2	7.2	1.0	2.0
146	1100	Handicap	Good	60.89	131.8	41.1	30.4	0.9	44.2	53.7	3.7
155	1100	Handicap	Good	62.14	83.8	22.1	55.7	0.3	10.2	1.3	3.0
156	1100	Handicap	Good	61.22	81.7	17.1	52.2	0.3	7.7	1.0	3.0
157	1100	Handicap	Good	63.73	81.7	17.1	52.2	0.3	7.7	1.0	3.0
158	1100	Handicap	Good	61.18	92.6	13.5	48.4	0.3	7.9	1.0	3.0
160	1100	Handicap	Good	61.26	98.8	14.3	43.5	0.2	8.7	1.0	3.2
161	1100	Handicap	Good	62.21	98.8	14.3	43.5	0.2	8.7	1.0	3.2
162	1100	Handicap	Good	60.88	108.2	16.4	38.9	0.3	10.1	1.0	3.2
163	1100	Handicap	Good	60.95	108.2	16.4	38.9	0.3	10.1	1.0	3.2
164	1100	Handicap	Good	61.24	82.9	24.4	25.0	0.3	15.5	12.2	2.5
168	1100	Handicap	Good	62.97	72.6	19.4	35.6	0.2	9.1	2.6	2.4
175	1100	Handicap	Good	63.83	70.1	18.8	38.9	0.2	5.3	1.0	2.1
265	1100	Handicap	Good	62.17	75.8	27.3	12.0	0.7	41.6	19.8	3.9
267	1100	Handicap	Good	61.62	74.3	28.9	11.1	0.7	45.2	16.6	2.9
268	1100	Handicap	Good	63.14	81.1	29.3	10.8	0.7	47.1	16.8	2.1
269	1100	Handicap	Good	63.06	81.1	29.3	10.8	0.7	47.1	16.8	2.1
270	1100	Handicap	Good	62.30	81.1	29.3	10.8	0.7	47.1	16.8	2.1

271	1100	Handicap	Good	62.56	81.3	28.7	9.9	0.8	46.9	17.9	1.4
272	1100	Handicap	Good	62.65	81.3	28.7	9.9	0.8	46.9	17.9	1.4
273	1100	Handicap	Good	62.41	80.4	28.4	9.4	0.9	45.0	33.6	1.9
274	1100	Handicap	Good	60.53	126.4	48.3		1.2	49.3	98.0	4.6
284	1100	Handicap	Good	62.64	116.6	41.6		0.6	49.4	22.7	3.6
285	1100	Handicap	Good	61.03	116.6	41.6		0.6	49.4	22.7	3.6
286	1100	Handicap	Good	64.80	119.6	38.7		0.6	47.6	13.7	3.4
287	1100	Handicap	Good	63.15	119.6	38.7		0.6	47.6	13.7	3.4
288	1100	Handicap	Good	62.06	119.6	38.7		0.6	47.6	13.7	3.4
289	1100	Handicap	Good	61.89	142.2	38.7		0.6	46.7	20.1	3.4
290	1100	Handicap	Good	60.81	142.2	38.7		0.6	46.7	20.1	3.4
291	1100	Handicap	Good	61.07	170.6	40.6		0.7	48.0	29.4	3.4
292	1100	Handicap	Good	60.71	170.6	40.6		0.7	48.0	29.4	3.4
311	1100	Handicap	Good	61.55	79.7	38.4	13.3	0.9	36.7	50.0	3.4
318	1100	Handicap	Good	63.32	67.9	30.1	35.5	0.6	34.3	9.8	3.3
324	1100	Handicap	Good	62.26	94.9	30.7	40.6	0.5	30.0	2.7	3.1
325	1100	Handicap	Good	62.02	94.9	30.7	40.6	0.5	30.0	2.7	3.1
326	1100	Handicap	Good	60.34	105.4	32.2	36.7	0.5	28.8	1.5	2.9
327	1100	Handicap	Good	60.12	105.4	32.2	36.7	0.5	28.8	1.5	2.9
328	1100	Handicap	Good	60.48	105.3	32.0	29.0	0.6	29.3	1.3	2.9
452	1100	Handicap	Good	61.48	125.8	48.9	8.2	1.1	79.7	70.1	2.5
453	1100	Handicap	Good	61.16	125.8	48.9	8.2	1.1	79.7	70.1	2.5
454	1100	Handicap	Good	63.06	113.3	40.8	8.1	0.9	70.3	42.1	2.3
455	1100	Handicap	Good	62.04	113.3	40.8	8.1	0.9	70.3	42.1	2.3
456	1100	Handicap	Good	61.88	107.0	35.8	7.2	0.8	62.2	29.8	2.3
457	1100	Handicap	Good	62.46	107.0	35.8	7.2	0.8	62.2	29.8	2.3
458	1100	Handicap	Good	61.24	107.0	35.8	7.2	0.8	62.2	29.8	2.3
459	1100	Handicap	Good	62.05	108.7	34.1	5.8	0.8	54.9	24.3	2.4

460	1100	Handicap	Good	62.43	108.7	34.1	5.8	0.8	54.9	24.3	2.4
461	1100	Handicap	Good	62.12	121.3	34.7	4.1	0.8	48.4	29.2	2.7
462	1100	Handicap	Good	61.33	121.3	34.7	4.1	0.8	48.4	29.2	2.7
463	1100	Handicap	Good	61.06	163.7	68.0	4.2	1.8	68.7	186.5	5.4
471	1100	Handicap	Good	63.93	133.5	51.3	15.1	0.9	70.8	45.9	4.7
473	1100	Handicap	Good	61.74	130.0	46.8	15.2	0.7	65.9	30.1	4.8
474	1100	Handicap	Good	61.56	130.0	46.8	15.2	0.7	65.9	30.1	4.8
475	1100	Handicap	Good	62.09	130.0	46.8	15.2	0.7	65.9	30.1	4.8
477	1100	Handicap	Good	62.76	123.8	40.4	14.1	0.6	58.9	20.4	4.8
478	1100	Handicap	Good	62.67	113.2	33.8	11.1	0.6	53.2	19.5	4.8
479	1100	Handicap	Good	62.61	113.2	33.8	11.1	0.6	53.2	19.5	4.8
480	1100	Handicap	Good	62.95	110.7	30.2	7.5	0.6	48.8	21.2	4.6
481	1100	Handicap	Good	63.36	110.7	30.2	7.5	0.6	48.8	21.2	4.6
492	1100	Handicap	Good	64.13	98.6	28.4	17.7	0.4	43.7	17.5	3.2
493	1100	Handicap	Good	62.13	98.6	28.4	17.7	0.4	43.7	17.5	3.2
494	1100	Handicap	Good	62.55	98.6	28.4	17.7	0.4	43.7	17.5	3.2
495	1100	Handicap	Good	62.31	100.0	27.8	16.9	0.3	40.0	9.0	2.9
496	1100	Handicap	Good	62.70	100.0	27.8	16.9	0.3	40.0	9.0	2.9
497	1100	Handicap	Good	62.62	101.9	28.5	14.6	0.2	38.0	5.6	2.6
498	1100	Handicap	Good	63.01	101.9	28.5	14.6	0.2	38.0	5.6	2.6
499	1100	Handicap	Good	62.61	100.9	30.6	11.3	0.2	38.5	4.7	2.5
500	1100	Handicap	Good	63.28	100.9	30.6	11.3	0.2	38.5	4.7	2.5
519	1100	Handicap	Good	60.56	64.4	23.0	5.8	0.7	25.0	67.0	2.7
521	1100	Handicap	Good	63.55	64.6	19.8	7.7	0.6	27.1	55.6	2.8
530	1100	Handicap	Good	60.76	49.1	8.8	11.8	0.3	29.9	16.1	2.5
531	1100	Handicap	Good	62.88	54.8	8.9	9.9	0.4	30.6	18.0	2.5
532	1100	Handicap	Good	61.27	54.8	8.9	9.9	0.4	30.6	18.0	2.5
533	1100	Handicap	Good	61.19	55.9	11.5	8.0	0.4	30.4	19.8	2.3

534	1100	Handicap	Good	62.47	55.9	11.5	8.0	0.4	30.4	19.8	2.3
535	1100	Handicap	Good	61.92	55.9	11.5	8.0	0.4	30.4	19.8	2.3
536	1100	Handicap	Good	62.17	58.6	14.3	6.0	0.5	30.3	27.3	2.2
555	1100	Handicap	Good	61.82	66.3	25.5	7.1	0.9	26.9	87.4	2.9
558	1100	Handicap	Good	63.82	44.1	14.0	12.4	0.5	21.3	37.8	1.9
568	1100	Handicap	Good	62.13	27.8	5.9	13.1	0.3	18.2	5.6	1.0
569	1100	Handicap	Good	61.48	27.8	5.9	13.1	0.3	18.2	5.6	1.0
570	1100	Handicap	Good	62.86	35.3	7.6	10.5	0.3	20.9	10.6	1.1
572	1100	Handicap	Good	62.18	48.3	10.5	7.8	0.4	23.1	20.4	1.3
573	1100	Handicap	Good	62.74	48.3	10.5	7.8	0.4	23.1	20.4	1.3
574	1100	Handicap	Good	62.11	74.9	38.7	10.5	1.1	29.2	57.8	2.4
585	1100	Handicap	Good	61.51	50.4	18.1	32.4	0.5	16.4	5.6	1.5
586	1100	Handicap	Good	62.42	49.0	14.7	33.8	0.4	11.8	2.6	1.6
587	1100	Handicap	Good	63.14	49.0	14.7	33.8	0.4	11.8	2.6	1.6
588	1100	Handicap	Good	61.66	46.4	13.6	32.1	0.4	10.1	1.3	1.6
589	1100	Handicap	Good	62.98	46.4	13.6	32.1	0.4	10.1	1.3	1.6
590	1100	Handicap	Good	63.45	46.4	13.6	32.1	0.4	10.1	1.3	1.6
591	1100	Handicap	Good	62.75	47.4	15.4	27.8	0.4	11.1	1.0	1.7
592	1100	Handicap	Good	63.11	47.4	15.4	27.8	0.4	11.1	1.0	1.7
12	1200	Handicap	Good	63.31	76.9	20.6	44.8	0.2	6.4	1.0	2.8
60	1200	Handicap	Good	62.87	66.1	16.3	27.3	0.2	7.9	3.0	2.0
73	1200	Handicap	Good	61.85	74.8	23.9	20.7	0.4	13.1	9.5	2.0
79	1200	Handicap	Good	61.36	82.6	20.1	32.4	0.2	8.8	2.3	2.0
80	1200	Handicap	Good	63.41	82.6	20.1	32.4	0.2	8.8	2.3	2.0
82	1200	Handicap	Good	62.85	81.8	20.2	33.8	0.2	7.7	1.5	2.0
84	1200	Handicap	Good	62.56	79.8	19.0	34.5	0.2	7.4	1.3	2.0
87	1200	Handicap	Good	61.74	78.8	20.8	34.7	0.2	7.8	1.2	2.1
88	1200	Handicap	Good	61.88	74.7	21.3	33.5	0.2	8.3	1.2	2.1

89	1200	Handicap	Good	62.01	74.7	21.3	33.5	0.2	8.3	1.2	2.1
90	1200	Handicap	Good	62.61	67.5	19.8	30.9	0.3	8.9	1.1	2.1
118	1200	Handicap	Good	63.10	55.4	11.5	37.0	0.2	6.5	1.3	2.1
137	1200	Handicap	Good	62.96	91.2	16.2	46.7	0.2	7.6	1.2	2.3
142	1200	Handicap	Good	62.29	114.2	20.2	38.1	0.2	6.4	1.0	2.0
145	1200	Handicap	Good	62.65	119.3	24.4	35.9	0.2	7.2	1.0	2.0
188	1200	Handicap	Good	62.59	56.7	21.1	35.7	0.2	10.0	3.5	1.4
199	1200	Handicap	Good	62.45	48.9	16.3	23.7	0.2	7.3	1.2	1.6
200	1200	Handicap	Good	63.15	48.9	16.3	23.7	0.2	7.3	1.2	1.6
215	1200	Handicap	Good	62.55	93.6	20.0	31.7	0.3	8.7	1.0	2.0
218	1200	Handicap	Good	62.00	86.5	19.9	28.9	0.3	8.9	1.0	2.0
246	1200	Handicap	Good	62.99	124.3	45.9	39.3	0.9	76.1	20.8	5.8
247	1200	Handicap	Good	62.43	127.9	44.0	41.5	0.8	69.9	13.2	5.3
251	1200	Handicap	Good	61.56	152.3	40.2	34.9	0.7	57.7	6.7	4.7
302	1200	Handicap	Good	62.79	86.5	27.1	15.5	0.5	46.5	33.3	3.3
303	1200	Handicap	Good	61.90	83.8	23.0	15.8	0.4	41.7	18.6	3.1
310	1200	Handicap	Good	61.79	120.8	27.2	8.6	0.5	38.6	19.2	3.1
337	1200	Handicap	Good	61.81	151.0	58.3		0.5	28.8	75.6	8.0
341	1200	Handicap	Good	61.70	156.6	63.6		0.8	30.5	93.0	8.6
468	1200	Handicap	Good	63.05	158.2	65.7	10.7	1.5	77.0	129.2	4.8
476	1200	Handicap	Good	62.14	123.8	40.4	14.1	0.6	58.9	20.4	4.8
564	1200	Handicap	Good	62.74	22.0	4.7	16.6	0.2	14.0	4.6	1.0
566	1200	Handicap	Good	62.10	23.9	4.9	15.5	0.2	15.4	4.4	1.0

Table S4. Dataset of "Conditional" races.

Number	Distance	Type of race	Track Condition	Speed	PM10	PM2.5	O3	CO	NO2	NO	SO2
7	1000	Conditional F2L	Good	61.27	73.1	22.4	44.3	0.2	6.8	1.1	2.3
259	1000	Conditional F2L	Good	61.38	67.3	24.3	8.9	0.8	35.3	35.1	4.0
260	1000	Conditional F2L	Good	61.05	67.3	24.3	8.9	0.8	35.3	35.1	4.0
312	1000	Conditional F2L	Good	61.04	80.1	37.2	19.4	0.8	38.7	35.6	3.5
314	1000	Conditional F2L	Good	61.92	73.8	34.3	27.7	0.7	37.7	17.0	3.4
315	1000	Conditional F2L	Good	61.39	73.8	34.3	27.7	0.7	37.7	17.0	3.4
206	1000	Conditional F3W	Good	64.67	91.9	22.6	33.7	0.3	8.0	1.4	1.9
470	1000	Conditional F3W	Good	63.42	146.8	59.4	13.1	1.2	75.9	83.2	4.8
148	1000	Conditional M-F3W	Good	61.43	104.9	36.0	40.3	0.7	37.9	34.5	3.4
83	1000	Conditional M-FW	Good	63.77	79.8	19.0	34.5	0.2	7.4	1.3	2.0
19	1000	Conditional M2L	Good	64.68	46.9	12.3	31.2	0.3	6.7	1.9	1.7
22	1000	Conditional M2L	Good	63.85	55.0	12.3	33.5	0.3	6.3	1.5	1.7
257	1000	Conditional M2L	Good	60.98	66.5	25.3	7.3	0.9	33.0	47.1	4.1
264	1000	Conditional M2L	Good	61.83	75.8	27.3	12.0	0.7	41.6	19.8	3.9
317	1000	Conditional M2L	Good	61.62	67.9	30.1	35.5	0.6	34.3	9.8	3.3
136	1000	Conditional M3	Good	63.21	91.2	16.2	46.7	0.2	7.6	1.2	2.3
450	1000	Conditional M3W	Good	61.10	136.2	55.8	7.5	1.4	83.8	105.4	3.1
42	1100	Conditional F2L	Good	63.47	94.0	23.1	46.3	0.3	9.8	1.6	1.8
45	1100	Conditional F2L	Good	63.56	99.2	23.9	47.8	0.3	9.0	1.2	1.8
95	1100	Conditional F2L	Good	60.77	86.9	18.3	37.4	0.3	13.0	4.1	3.0
98	1100	Conditional F2L	Good	61.55	82.8	16.9	40.7	0.3	10.3	2.3	2.9
149	1100	Conditional F2L	Good	61.03	89.3	30.8	48.5	0.5	26.8	13.0	3.2
152	1100	Conditional F2L	Good	60.94	84.6	26.3	54.4	0.3	15.5	3.1	3.0
520	1100	Conditional F2L	Good	61.45	64.6	19.8	7.7	0.6	27.1	55.6	2.8
523	1100	Conditional F2L	Good	61.96	49.4	12.2	9.8	0.5	28.2	34.7	2.4

59	1100	Conditional F3W	Good	63.08	66.1	16.3	27.3	0.2	7.9	3.0	2.0
2	1100	Conditional F3L	Good	62.99	84.0	28.5	34.1	0.3	15.4	11.1	2.4
21	1100	Conditional F3L	Good	62.58	55.0	12.3	33.5	0.3	6.3	1.5	1.7
46	1100	Conditional F3L	Good	62.38	104.4	24.3	47.0	0.3	8.3	1.1	1.9
64	1100	Conditional F3L	Good	62.54	64.7	15.8	31.7	0.2	5.8	1.2	2.0
86	1100	Conditional F3L	Good	61.68	78.8	20.8	34.7	0.2	7.8	1.2	2.1
92	1100	Conditional F3L	Good	63.22	92.0	20.8	31.6	0.4	16.5	9.3	3.1
111	1100	Conditional F3L	Good	62.77	60.6	16.3	27.7	0.3	13.5	10.9	2.1
129	1100	Conditional F3L	Good	61.94	82.3	21.6	35.4	0.5	28.4	20.9	3.0
159	1100	Conditional F3L	Good	61.31	92.6	13.5	48.4	0.3	7.9	1.0	3.0
165	1100	Conditional F3L	Good	61.92	71.8	20.9	30.9	0.2	12.0	5.2	2.5
187	1100	Conditional F3L	Good	63.11	60.5	22.3	35.7	0.3	10.9	4.0	1.4
210	1100	Conditional F3L	Good	60.59	89.1	20.0	35.9	0.3	7.6	1.3	2.0
283	1100	Conditional F3L	Good	61.41	121.5	45.8		0.7	52.4	38.3	4.0
321	1100	Conditional F3L	Good	62.05	84.3	29.7	42.6	0.5	30.4	3.9	3.2
336	1100	Conditional F3L	Good	61.04	141.1	52.5		0.4	27.4	67.4	7.7
488	1100	Conditional F3L	Good	62.94	106.8	35.9	14.4	0.8	56.6	71.2	4.2
491	1100	Conditional F3L	Good	62.86	101.3	31.3	17.2	0.5	48.5	34.1	3.6
116	1100	Conditional F3W	Good	63.79	53.4	12.3	36.5	0.2	8.1	2.0	2.1
248	1100	Conditional M-FL	Good	62.16	127.9	44.0	41.5	0.8	69.9	13.2	5.3
131	1100	Conditional M2L	Good	61.26	83.5	19.8	41.1	0.4	21.2	10.5	2.8
134	1100	Conditional M2L	Good	63.05	85.8	16.9	45.6	0.3	12.0	3.0	2.5
522	1100	Conditional M2L	Good	61.40	49.4	12.2	9.8	0.5	28.2	34.7	2.4
524	1100	Conditional M2L	Good	61.83	46.6	8.4	11.6	0.4	28.6	26.8	2.0
6	1100	Conditional M3L	Good	62.67	77.2	24.4	40.4	0.3	9.8	3.3	2.3
25	1100	Conditional M3L	Good	62.20	64.8	12.1	34.8	0.3	6.0	1.3	1.8
37	1100	Conditional M3L	Good	62.62	87.6	23.5	38.0	0.3	14.7	5.8	1.9
56	1100	Conditional M3L	Good	62.51	65.7	16.3	22.9	0.2	8.9	4.5	2.0

58	1100	Conditional M3L	Good	62.71	66.1	16.3	27.3	0.2	7.9	3.0	2.0
74	1100	Conditional M3L	Good	62.20	79.0	22.7	25.4	0.3	11.3	5.8	2.0
78	1100	Conditional M3L	Good	61.40	80.8	21.6	29.5	0.3	10.0	3.5	2.0
85	1100	Conditional M3L	Good	61.07	78.8	20.8	34.7	0.2	7.8	1.2	2.1
102	1100	Conditional M3L	Good	61.99	81.8	15.8	39.8	0.2	6.7	1.0	2.6
113	1100	Conditional M3L	Good	62.38	56.0	13.8	32.5	0.3	10.6	4.9	2.1
114	1100	Conditional M3L	Good	62.41	56.0	13.8	32.5	0.3	10.6	4.9	2.1
135	1100	Conditional M3L	Good	60.92	85.8	16.9	45.6	0.3	12.0	3.0	2.5
147	1100	Conditional M3L	Good	60.72	104.9	36.0	40.3	0.7	37.9	34.5	3.4
183	1100	Conditional M3L	Good	62.20	61.6	24.6	28.7	0.4	15.1	15.1	1.6
205	1100	Conditional M3L	Good	62.15	92.4	26.8	28.9	0.4	20.8	16.8	1.9
282	1100	Conditional M3L	Good	61.62	121.5	45.8		0.7	52.4	38.3	4.0
338	1100	Conditional M3L	Good	62.29	151.0	58.3		0.5	28.8	75.6	8.0
483	1100	Conditional M3L	Good	61.49	162.4	47.4	6.8	1.4	65.9	155.9	5.2
486	1100	Conditional M3L	Good	61.26	117.3	41.0	10.7	1.1	63.6	116.6	4.8
3	1100	Conditional M3W	Good	63.10	84.0	28.5	34.1	0.3	15.4	11.1	2.4
484	1100	Conditional F3L	Good	62.89	162.4	47.4	6.8	1.4	65.9	155.9	5.2
13	1100	Conditional F3W	Good	64.04	80.0	20.3	42.5	0.2	6.9	1.0	2.8
39	1100	Conditional M3L	Good	62.67	90.9	23.3	43.6	0.3	12.2	2.8	1.8
445	1200	Conditional F3L	Good	61.64	167.9	67.5	4.3	2.0	78.7	200.0	4.6
186	1200	Conditional F2L	Good	61.85	60.5	22.3	35.7	0.3	10.9	4.0	1.4
275	1200	Conditional F2L	Good	62.31	136.3	51.6		1.2	54.7	88.3	4.6
330	1200	Conditional F2L	Good	61.85	116.6	44.5		0.2	23.8	45.4	6.8
334	1200	Conditional F2L	Good	62.04	126.6	47.8		0.3	25.5	54.5	7.2
240	1200	Conditional F2W	Good	62.18	148.0	57.2	21.5	1.5	90.9	97.4	7.0
261	1200	Conditional F2W	Good	63.78	70.0	24.9	10.9	0.7	37.2	25.0	3.9
189	1200	Conditional F2L	Good	61.65	56.7	21.1	35.7	0.2	10.0	3.5	1.4
28	1200	Conditional F3W	Good	63.23	95.4	11.8	32.6	0.3	5.4	1.1	1.9

77	1200	Conditional F3W	Good	62.74	80.8	21.6	29.5	0.3	10.0	3.5	2.0
451	1200	Conditional F3L	Good	62.53	136.2	55.8	7.5	1.4	83.8	105.4	3.1
166	1200	Conditional M2L	Good	62.79	71.8	20.9	30.9	0.2	12.0	5.2	2.5
167	1200	Conditional M2L	Good	62.61	72.6	19.4	35.6	0.2	9.1	2.6	2.4
202	1200	Conditional M2L	Good	62.61	93.8	29.8	23.4	0.5	23.9	26.6	1.9
204	1200	Conditional M2L	Good	62.45	92.4	26.8	28.9	0.4	20.8	16.8	1.9
238	1200	Conditional M2L	Good	62.19	163.3	61.0	13.6	1.9	90.7	147.4	7.6
241	1200	Conditional M2L	Good	62.12	148.0	57.2	21.5	1.5	90.9	97.4	7.0
294	1200	Conditional M2L	Good	63.42	114.3	45.8	7.4	1.4	56.9	155.2	4.9
296	1200	Conditional M2L	Good	61.69	96.5	37.1	10.3	1.1	55.1	110.3	4.3
40	1200	Conditional M3W	Good	63.32	90.9	23.3	43.6	0.3	12.2	2.8	1.8
464	1200	Conditional M3L	Good	62.39	170.4	70.1	7.1	1.8	75.3	168.1	5.2
467	1200	Conditional M3L	Good	63.77	158.2	65.7	10.7	1.5	77.0	129.2	4.8
278	1200	Conditional F2L	Good	61.90	136.3	51.2		1.1	56.7	71.3	4.5

The code for the different types of "Conditional" races is read as follows: first there is the gender (M=male/F=female), age and previous sports results (L=losers, W=winners).
